# Improved Adaptive Sliding Mode Control Using Quasi-Convex Functions and Neural Network-Assisted Time-Delay Estimation for Robotic Manipulators

**DOI:** 10.3390/s25144252

**Published:** 2025-07-08

**Authors:** Jin Woong Lee, Jae Min Rho, Sun Gene Park, Hyuk Mo An, Minhyuk Kim, Seok Young Lee

**Affiliations:** 1 Department of ICT Convergence Engineering, Soonchunhyang University, Asan 31538, Republic of Korea; 2 Department of Electronic Engineering, Soonchunhyang University, Asan 31538, Republic of Korea; 3 Department of Artificial Intelligence and Information Technology, Sejong University, Seoul 05006, Republic of Korea

**Keywords:** robotic manipulator, adaptive sling mode control, uniform ultimate boundedness, time-delay control, neural networks

## Abstract

This study presents an adaptive sliding mode control strategy tailored for robotic manipulators, featuring a quasi-convex function-based control gain and a time-delay estimation (TDE) enhanced by neural networks. To compensate for TDE errors, the proposed method utilizes both the previous TDE error and radial basis function neural networks with a weight update law that includes damping terms to prevent divergence. Additionally, a continuous gain function that is quasi-convex function dependent on the magnitude of the sliding variable is proposed to replace the traditional switching control gain. This continuous function-based gain has effectiveness in suppressing chattering phenomenon while guaranteeing the stability of the robotic manipulator in terms of uniform ultimate boundedness, which is demonstrated through both simulation and experiment results.

## 1. Introduction

Robotic manipulators have been increasingly employed across various fields, including manufacturing [1], surgery [2], service industries [3], and agriculture [4], due to their high versatility. To operate effectively across diverse fields, robotic manipulators must exhibit high accuracy and reliability, which requires precise model information for their nonlinear dynamics. However, highly nonlinear properties, external disturbances, and inaccuracies in system modeling make it difficult to obtain precise model information. Consequently, researchers have explored diverse control approaches including time-delay control (TDC) [5,6,7,8] and sliding mode control (SMC) [5,9,10,11,12,13] to cope with these issues.

In real environments, obtaining exact dynamic equations of robotic manipulators is challenging work due to inaccuracies in system modeling and external disturbances. Thus, a TDC technique utilizes time-delay estimation (TDE) to approximate unpredictable disturbances and inaccuracies in system modeling of robotic manipulators [5,6,7,8]. The TDE uses previous accelerations and control torques for estimation because it is based on the assumption that an estimated value is the same as before when the sampling period is small enough. However, the sampling period cannot be reduced indefinitely due to limitations in communication speeds and hardware performance, which leads to TDE errors [5]. In [8], while an enhanced TDE structure was proposed to reduce the current TDE error by utilizing the previous one scaled by a tunable parameter, this parameter increase control complexity. Also, TDE errors have not been completely eliminated, requiring an additional compensation strategy. To better handle TDE errors, methods combining TDC and SMC were proposed in [5,8]. Further, neural networks (NNs) have been utilized with robust control strategies [14,15,16,17].

SMC is a robust control scheme widely recognized for its simplicity and effectiveness [9,10]. To compensate for TDE errors, SMC input utilizes an adjustable gain, which steers the sliding variable toward the sliding surface whenever the control gain exceeds the total system uncertainties. However, it is difficult work to precisely determine SMC gain satisfying such condition. Thus, adaptive sliding mode control (ASMC) that adjusts the SMC gain using an adaptive law dependent on the sliding variable has been proposed [5,11,12,13]. Generally, the adaptive update law raises SMC gain for the convergence of the sliding variable toward zero [11]. However, in real environments, the sliding variable can never become exactly zero [5,12], which leads to an overestimated ASMC gain and increased chattering phenomenon. To address this issue, adaptive laws have been proposed that reduce ASMC gain when the sliding variable converges to a specific region and increase it when the sliding variable deviates from this region [8,12]. However, adaptive laws that switch at specific boundaries may exacerbate chattering phenomenon due to an abrupt change in the control gain. Thus, a class $K_{\infty }$ function-based gain that are convex or concave with respect to a magnitude of the sliding variable was proposed in [13]. Although the work [13] shows that the convex function-based gain outperforms the concave one in terms of control performance, the steep gradient of the convex function may result in an overestimated gain with exacerbated chattering phenomenon.

NNs have been actively explored in control system design [14,15,16,17,18,19] due to their ability to estimate unknown parameters by utilizing input–output data. In the works [14,15], NNs estimate the nonlinear dynamics and approximate overall uncertainties with the SMC scheme. In [16,17], NNs are employed to adaptively tune SMC gains. In [18,19], NNs are integrated with TDC to estimate inaccuracies in system modeling or approximate TDE errors. In particular, [18] introduced a radial basis function neural network (RBFNN) to estimate TDE errors. Although these studies demonstrated that the RBFNN enhances the control performance of TDC, they were limited to simulations and lacked consideration of applicability to robotic manipulators in real environments.

Based on the aforementioned discussions, this paper proposes an ASMC strategy tailored for robotic manipulators, featuring a quasi-convex function-based control gain and the TDE enhanced by RBFNN. The main contributions of this paper are summarized as follows.

(1) To compensate for TDE errors, the proposed method utilizes both the previous TDE error and the RBFNN with a weight update law that includes damping terms to prevent divergence.
(2) A continuous gain, designed as a quasi-convex function with respect to the magnitude of the sliding variable, is proposed to replace the traditional switching adaptive law. This function preserves the continuous gain and smooth transitions between convex and concave characteristics depending on the magnitude of the sliding variable.
(3) The stability of the proposed control method is guaranteed in the sense of uniform ultimate boundedness, and its effectiveness is validated through both simulation and experiment results.

This paper is organized as follows. Section 2 introduces the system dynamics and preliminary definitions required for control design. Section 3 presents the proposed TDE enhanced by RBFNN and the quasi-convex function-based ASMC. In Section 4, simulation results on a 2-joint robotic manipulator are provided. Section 5 validates the performance of the proposed method through experiments on a real robotic manipulator. Finally, Section 6 concludes the study.

*Notations*: R, $R^{n}$, and $R^{n\times m}$ denote the set of real numbers, *n*-dimensional real vectors, and n×m real matrices, respectively. diag{·} denotes a diagonal matrix with the indicated elements. $I_{n}$ denotes an $R^{n\times n}$ identity matrix. $J_{n,m}$ denotes a matrix in $R^{n\times m}$ with entries that are all equal to one. sgn(·) represents the signum function. ${∥x\left(t\right)∥}_{\infty }$ and ∥x(t)∥ denote the infinity norm and the Euclidean norm of the vector x(t), respectively.

## 2. Preliminaries

**Definition** **1**  ([20])**.** *Given the sliding variable σ and a positive scalar δ, the real sliding surface is defined as*

(1) $$\begin{array}{c} \Sigma =\left\{\sigma \in R^{n}|{∥\sigma ∥}_{\infty }\leq \delta \right\}. \end{array}$$

**Definition** **2**  ([21])**.** *A continuous function β:[0,c)→[0,∞) is considered a class K function if it is strictly increasing and β(0)=0. If the domain [0,c) is extended to [0,∞) and β(s)→∞ as s→∞, then β belongs to class $K_{\infty }$ function.*

**Lemma** **1**  ([21])**.** *Let $\beta_{1}$ be a class K function defined on [0,c), and $\beta_{2}$ be a class $K_{\infty }$ function. Define the inverse of $\beta_{i}$ as $\beta_{i}^{-1}$, i=1,2. Then, the following statements hold:*

- *$\beta_{1}^{-1}$ is defined on $\left[0,\beta_{1}\left(c\right)\right)$ and belongs to class K.*
- *$\beta_{2}^{-1}$ is defined on [0,∞) and belongs to class $K_{\infty }$.*

The dynamic equation of a robotic manipulator with *n*-joint under external disturbances can be described by the following expression.

(2) $$\begin{array}{c} H\left(\theta \left(t\right)\right)\ddot{\theta }\left(t\right)+C\left(\theta \left(t\right),\dot{\theta }\left(t\right)\right)\dot{\theta }\left(t\right)+G\left(\theta \left(t\right)\right)+F\left(\dot{\theta }\left(t\right)\right)=\tau \left(t\right)+\tau_{d}\left(t\right). \end{array}$$

Here, θ(t), $\dot{\theta }\left(t\right)$, and $\ddot{\theta }\left(t\right)$ are vectors representing the positions, velocities, and accelerations of the joints, respectively, in $R^{n}$. H(θ(t)) is the inertia matrix, and $C(\theta \left(t\right),\dot{\theta }\left(t\right))$ is the Coriolis matrix, in $R^{n\times n}$. The gravity force vector $G\left(\theta \left(t\right)\right)\in R^{n}$ and the friction force vector $F\left(\dot{\theta }\left(t\right)\right)\in R^{n}$ account for gravitational and frictional effects acting on the robot joints. The control torque $\tau \left(t\right)\in R^{n}$ and the unpredictable disturbance $\tau_{d}\left(t\right)\in R^{n}$ influence the system dynamics. By multiplying both sides of the Equation (2) by $H^{-1}\left(\theta \left(t\right)\right)$ and reorganizing it with respect to the acceleration $\ddot{\theta }\left(t\right)$, the Equation (2) can be expressed as

(3) $$\begin{array}{c} \ddot{\theta }\left(t\right)={\bar{H}}^{-1}\tau \left(t\right)+N\left(t\right). \end{array}$$

Here, $\bar{H}=diag\left\{{\bar{h}}_{1},{\bar{h}}_{2},\ldots ,{\bar{h}}_{n}\right\}\in R^{n\times n}$ represents a gain matrix and N(t) is given below.

(4) $$\begin{array}{c} N\left(t\right)=-{\bar{H}}^{-1}\left\{C\left(\theta \left(t\right),\dot{\theta }\left(t\right)\right)\dot{\theta }\left(t\right)+G\left(\theta \left(t\right)\right)+F\left(\dot{\theta }\left(t\right)\right)-\tau_{d}\left(t\right)\right\}-{\bar{H}}^{-1}\left(H\left(\theta \left(t\right)\right)-\bar{H}\right)\ddot{\theta }\left(t\right). \end{array}$$

It is difficult to precisely derive N(t) in real environments, as it exhibits nonlinear and time-varying characteristics. If the sampling period *L* is sufficiently small, N(t) can be expressed using the TDE as follows.

(5) $$\begin{array}{c} N\left(t\right)\approx \hat{N}\left(t\right)=N\left(t-L\right)=\ddot{\theta }\left(t-L\right)-{\bar{H}}^{-1}\tau \left(t-L\right). \end{array}$$

Using the TDE method (5), the control input $\tau_{1}^{TDC}\left(t\right)$ aiming to track the desired position $\theta_{d}\left(t\right)$ is expressed by

(6) $$\begin{array}{c} \tau_{1}^{TDC}\left(t\right)=\bar{H}\left({\ddot{\theta }}_{d}\left(t\right)+\left(\ell_{1}+\ell_{2}\right)\dot{e}\left(t\right)+\ell_{1}\ell_{2}e\left(t\right)\right)-\bar{H}N\left(t-L\right), \end{array}$$

where $\ell_{1},\ell_{2}$ are positive scalars and $e\left(t\right)=\theta_{d}\left(t\right)-\theta \left(t\right)$ is a tracking error. Substituting (5) and (6) into (3), the following error dynamic equation can be obtained.

(7) $$\begin{array}{c} \ddot{e}\left(t\right)+\left(\ell_{1}+\ell_{2}\right)\dot{e}\left(t\right)+\ell_{1}\ell_{2}e\left(t\right)+\phi \left(t\right)=0, \end{array}$$

where ϕ(t)≜N(t)−N(t−L) is the TDE error. Since the estimation error depends on *L*, the TDE error ϕ(t) is small and bounded when *L* is approximately zero as follows.

(8) $$\begin{array}{c} {∥\phi \left(t\right)∥}_{\infty }\leq \phi^{*}, \end{array}$$

where $\phi^{*}$ is a positive constant [8]. In order to mitigate the current TDE error ϕ(t), the work [8] introduces an improved TDC control input $\tau_{2}^{TDC}\left(t\right)$ defined as

(9) $$\begin{array}{c} \tau_{2}^{TDC}\left(t\right)=\bar{H}\left({\ddot{\theta }}_{d}\left(t\right)+\left(\ell_{1}+\ell_{2}\right)\dot{e}\left(t\right)+\ell_{1}\ell_{2}e\left(t\right)\right)-\bar{H}\left(N\left(t-L\right)+\alpha \phi \left(t-L\right)\right), \end{array}$$

where α is a tunable scalar. Then, substituting the TDC control torque $\tau_{2}^{TDC}\left(t\right)$ into the Equation (3), the following error dynamic equation can be obtained as follows.

(10) $$\begin{array}{c} \ddot{e}\left(t\right)+\left(\ell_{1}+\ell_{2}\right)\dot{e}\left(t\right)+\ell_{1}\ell_{2}e\left(t\right)+\phi \left(t\right)-\alpha \phi \left(t-L\right)=0, \end{array}$$

where ϕ(t−L) denotes a previous TDE error. In the Equation (10), the current TDE error ϕ(t) can be reduced by the previous TDE error ϕ(t−L) if the tunable parameter α is properly selected. Then, to compensate for the following novel TDE error,

(11) $$\begin{array}{c} \overset{˘}{\phi }\left(t\right)=\phi \left(t\right)-\alpha \phi \left(t-L\right), \end{array}$$

the ASMC torque $\tau_{1}^{ASMC}\left(t\right)$ is given by the equation below.

(12) $$\begin{array}{cc} \tau_{1}^{ASMC}\left(t\right)= & \bar{H}\left({\ddot{\theta }}_{d}\left(t\right)+\ell_{1}\dot{e}\left(t\right)+\ell_{2}\sigma \left(t\right)\right)\\ \; & -\bar{H}\left(N\left(t-L\right)+\alpha \phi \left(t-L\right)\right)+\bar{H}K\left(\sigma \left(t\right)\right)sgn\left(\sigma \left(t\right)\right). \end{array}$$

In this expression, $\sigma \left(t\right)=\dot{e}\left(t\right)+\ell_{1}e\left(t\right)$ is the sliding variable, and $K\left(\sigma \left(t\right)\right)\in R^{n\times n}$ denotes an ASMC gain matrix associated with the sliding variable.

## 3. Proposed ASMC and TDE Enhanced by NNs

**Figure 1:**
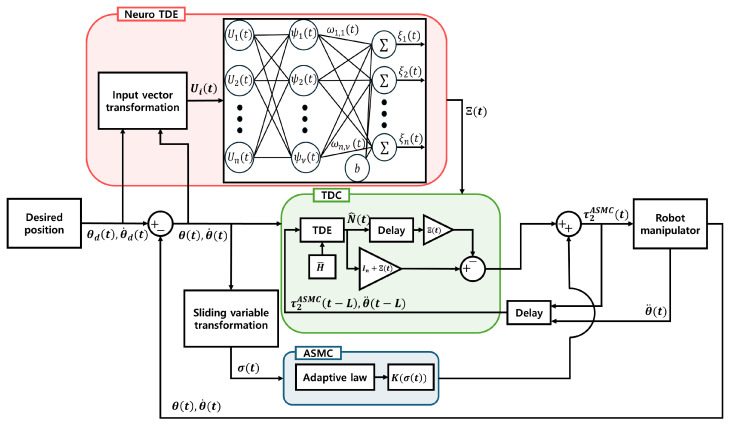
Block diagram illustrating the structure of the proposed methods.

A quasi-convex function-based ASMC and a TDE enhanced by NNs are proposed in this paper, as illustrated in Figure 1. We have the following ASMC input.

(13) $$\begin{array}{cc} \tau_{2}^{ASMC}\left(t\right)= & \bar{H}\left({\ddot{\theta }}_{d}\left(t\right)+\ell_{1}\dot{e}\left(t\right)+\ell_{2}\sigma \left(t\right)\right)\\ \; & -\bar{H}\left(N\left(t-L\right)+\Xi \left(t\right)\phi \left(t-L\right)\right)+\bar{H}K\left(\sigma \left(t\right)\right)sgn\left(\sigma \left(t\right)\right). \end{array}$$

When the ASMC (13) is utilized in place of the existing ASMC (12), the TDE error (11) transforms into the following TDE error:

(14) $$\begin{array}{c} \tilde{\phi }\left(t\right)=\phi \left(t\right)-\Xi \left(t\right)\phi \left(t-L\right). \end{array}$$

In this formulation, $\Xi \left(t\right)=diag\left\{\xi_{1}\left(t\right),\xi_{2}\left(t\right),\ldots ,\xi_{n}\left(t\right)\right\}$ corresponds to the output of the proposed RBFNN, and $K\left(\sigma \left(t\right)\right)=diag\left\{k_{1}\left(\sigma_{1}\left(t\right)\right),k_{2}\left(\sigma_{2}\left(t\right)\right),\ldots ,k_{n}\left(\sigma_{n}\left(t\right)\right)\right\}$. The proposed RBFNN architecture includes an input layer, a hidden layer with nonlinear activation functions, and an output layer. Its structure can be described as follows.

(15) $$\begin{array}{c} \xi_{i}\left(t\right)=\sum_{j=1}^{\nu }\omega_{i,j}\left(t\right)\psi_{j}\left(U_{i}\left(t\right)\right)+b. \end{array}$$

Here, ν denotes the number of hidden units. $\omega_{i,j}\left(t\right)$, $U_{i}\left(t\right)$, and *b* represent the weights, the input vector, and the bias, respectively, for i=1,…,n and j=1,…,ν. The activation function $\psi_{j}\left(U_{i}\left(t\right)\right)$ is constructed using a Gaussian RBF, formulated as

(16) $$\begin{array}{c} \psi_{j}\left(U_{i}\left(t\right)\right)=\exp\left(-\frac{d{\left(U_{i}\left(t\right),M_{j}\right)}^{2}}{2\eta^{2}}\right), \end{array}$$

where $U_{i}\left(t\right)=\left[\begin{array}{ccc} e_{i}\left(t\right) & \theta_{d,i}\left(t\right) & {\dot{\theta }}_{d,i}\left(t\right) \end{array}\right]$ is defined as an input vector containing the tracking error, the desired position, and its velocity of the *i*-th joint. $d(U_{i}\left(t\right),M_{j})$ represents the squared Euclidean distance between the input vector $U_{i}\left(t\right)$ and the center vector $M_{j}$, given by $∥U_{i}\left(t\right)-M_{j}∥$. Here, $M_{j}=\left[\begin{array}{ccc} m_{j,1} & m_{j,2} & m_{j,3} \end{array}\right]$ is the center vector of the Gaussian RBF, and η denotes its width parameter. The parameter η determines the width of activation range of the basis function. A smaller η results in a steeper variation of the function value near the center. The weight $\omega_{i,j}\left(t\right)$ is updated using the following update law.

(17) $$\begin{array}{c} {\dot{\omega }}_{i,j}\left(t\right)=\zeta_{i}\left|\sigma_{i}\left(t\right)\right|\psi_{j}\left(U_{i}\left(t\right)\right)-\gamma_{i}\omega_{i,j}, \end{array}$$

where $\zeta_{i}$ and $\gamma_{i}$ are positive scalars.

**Remark** **1.** *Compared to the TDE in [8], which utilizes a fixed parameter α in* (10), *the TDE enhanced by NNs* (13) *utilizes tracking errors, desired positions, and their derivative as input data, enabling the estimation of Ξ(t) for the current pose of robotic manipulators. Additionally, compared to those in [18,22], the proposed RBFNN incorporates a damping term in its weight update law* (17) *to prevent weight divergence. The effectiveness of the proposed TDE is shown through both simulation and experiment results.*

The proposed function-based gain is defined as follows.

(18) $$\begin{array}{c} k_{i}\left(\sigma_{i}\left(t\right)\right)=\rho \ln\left(1+{\left(\frac{\sigma_{i}\left(t\right)}{\lambda }\right)}^{2}\right), \end{array}$$

where ρ and λ are positive tuning parameters. The parameter ρ determines the overall scaling of the control gain. The parameter λ serves as a normalization factor that determines the degree to which the gain responds to the sliding variable. A smaller λ yields a steeper gain increase for small errors, while a larger λ results in a more gradual change. The gain function $k_{i}\left(\sigma_{i}\left(t\right)\right)$ belongs to class $K_{\infty }$ in Definition 2 and satisfies the following properties:

- The $k_{i}\left(\sigma_{i}\left(t\right)\right)$ is continuous on [0,∞).
- The $k_{i}\left(\sigma_{i}\left(t\right)\right)$ is at least $C^{0}$ with respect to $|\sigma_{i}\left(t\right)|$ on (0,∞).
- The inverse function of $k_{i}\left(\sigma_{i}\left(t\right)\right)$ exists.
- As $\sigma_{i}\left(t\right)$ approaches *∞*, the $k_{i}\left(\sigma_{i}\left(t\right)\right)$ approaches *∞*.

**Figure 2:**
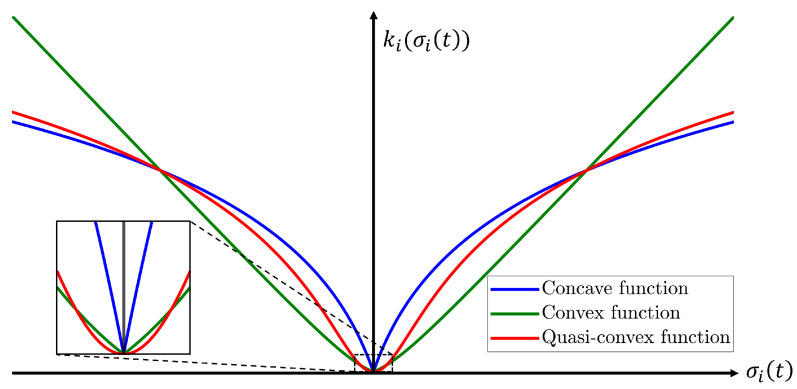
Comparison of class $K_{\infty }$ functions.

**Remark** **2.** *The continuous gain function* (18) *that is quasi-convex with respect to the magnitude of the sliding variable is proposed to replace the traditional switching control gain. The traditional switching-based adaptive law suffers from the drawback of inducing chattering phenomenon due to the discontinuous variation of the control gain. In contrast, the proposed function is a continuous function that belongs to class $K_{\infty }$, allowing the control gain to be adjusted smoothly and continuously according to the magnitude of the sliding variable, thereby effectively mitigating this issue. Also, this function preserves smooth transitions between convex and concave characteristics depending on the magnitude of the sliding variable. As illustrated in Figure 2, a convex function has a steep gradient as the sliding variable increases, whereas a concave one has a steep gradient near the origin but becomes flatter as the sliding variable increases. Therefore, convex and concave function-based gains have drawbacks at large sliding variable values and near the origin, respectively. In contrast, the proposed quasi-convex function-based gain is convex near the origin and concave as the sliding variable increases. The effectiveness of the proposed function is verified via tracking performance and chattering phenomenon observed in the control torque.*

To investigate the stability of the system (2) under the ASMC (13), the following Lyapunov function is introduced.

(19) $$\begin{array}{c} L\left(\sigma \left(t\right)\right)=\frac{1}{2}\sigma^{T}\left(t\right)\sigma \left(t\right). \end{array}$$

Using the Lyapunov function (19), the main result can be formulated as follows.

**Theorem** **1.** *For positive constants ρ, λ, and any initial sliding variable σ(0), if the proposed control torque* (13) *is applied to the system* (2), *then the sliding variable $\sigma \left(t\right)\in R^{n}$ is uniformly ultimately bounded in the sense that*

(20) $$\begin{array}{c} {∥\sigma \left(t\right)∥}_{\infty }\leq \delta_{\max}, \end{array}$$

*where*

(21) $$\begin{array}{cc} \delta_{\max}≜ & \max_{1\leq i\leq n}\left\{\delta_{i}\right\},\;i=1,2,\ldots ,n, \end{array}$$

(22) $$\begin{array}{cc} \delta_{i}= & \lambda \sqrt{\exp\left(\frac{{\bar{\phi }}_{i}}{\rho }\right)-1}. \end{array}$$

*Here, $\tilde{\phi }\left(t\right)={\left[\begin{array}{cccc} {\tilde{\phi }}_{1}\left(t\right) & {\tilde{\phi }}_{2}\left(t\right) & \cdots & {\tilde{\phi }}_{n}\left(t\right) \end{array}\right]}^{T}$ denotes the TDE error and ${\bar{\phi }}_{i}$ presents an upper bound of ${\tilde{\phi }}_{i}\left(t\right)$ in *(14)*.*

**Proof.**  The derivative of the Lyapunov function (19) with respect to time is computed as follows.

(23) $$\begin{array}{cc} \frac{d}{dt}L\left(\sigma \left(t\right)\right)= & \sigma^{T}\left(t\right)\dot{\sigma }\left(t\right)\\ = & \sigma^{T}\left(t\right)\left({\ddot{\theta }}_{d}\left(t\right)+\ell_{1}\dot{e}\left(t\right)-{\bar{H}}^{-1}\tau_{2}^{ASMC}\left(t\right)-\hat{N}\left(t\right)\right)\\ = & \sum_{i=1}^{n}\left\{\left(-\ell_{2}\sigma_{i}\left(t\right)-\phi_{i}\left(t\right)+\xi_{i}\left(t\right)\phi_{i}\left(t-L\right)-k_{i}\left(\sigma_{i}\left(t\right)\right)sgn\left(\sigma_{i}\left(t\right)\right)\right)\sigma_{i}\left(t\right)\right\}\\ = & \sum_{i=1}^{n}\{-\ell_{2}\sigma_{i}^{2}\left(t\right)-{\tilde{\phi }}_{i}\left(t\right)\sigma_{i}\left(t\right)-k_{i}\left(\sigma_{i}\left(t\right)\right)|\sigma_{i}\left(t\right)\left|\right\}\\ \leq & \sum_{i=1}^{n}\{-\ell_{2}\sigma_{i}^{2}\left(t\right)-\left(k_{i}\left(\sigma_{i}\left(t\right)\right)-{\bar{\phi }}_{i}\right)|\sigma_{i}\left(t\right)\left|\right\}. \end{array}$$

The derivative of the Lyapunov function (19) with respect to time is guaranteed to be negative if the gain function satisfies $k_{i}\left(\sigma_{i}\left(t\right)\right)>{\bar{\phi }}_{i}$. Since $k_{i}\left(\sigma_{i}\left(t\right)\right)$ is strictly increasing and belongs to class $K_{\infty }$, its inverse $k_{i}^{-1}\left(\sigma_{i}\left(t\right)\right)$ also exists and belongs to the same class, as established in Lemma 1. Accordingly, one can define the threshold $\delta_{i}$ such that $k_{i}\left(\delta_{i}\right)={\bar{\phi }}_{i}$, which yields $\delta_{i}=k_{i}^{-1}\left({\bar{\phi }}_{i}\right)$. Thus, if $|\sigma_{i}\left(t\right)|>\delta_{i}$ for each joint, the inequality $k_{i}\left(\sigma_{i}\left(t\right)\right)>{\bar{\phi }}_{i}$ holds, ensuring that $\frac{d}{dt}L\left(\sigma \left(t\right)\right)<0$. This result guarantees that $\sigma_{i}\left(t\right)$ converges to within the bound $\delta_{i}$. □

**Remark** **3.** *In the works [5,8], ASMC gain is updated according to the infinity norm of the sliding variable. Since such an adaptive law is mainly sensitive to the largest-magnitude element in the sliding variable or in the TDE error, there may exist conservative gain selection for the other joints except for the joint associated with the largest-magnitude element. In contrast, the proposed quasi-convex function-based gain tailored to individual joint behavior enables less conservative gain selection and individual bound* (22) *corresponding to each element of the sliding variable.*

## 4. Simulation

### 4.1. Simulation Setup

The simulation is performed based on the dynamic model (2) of a 2-joint robotic manipulator, given by

(24) $$\begin{array}{cc} H(\theta (t)) & =\left[\begin{array}{cc} l_{2}^{2}h_{2}+2\left(l_{1}l_{2}h_{2}\right)cos\left(\theta_{2}\left(t\right)\right)+l_{1}^{2}\left(h_{1}+h_{2}\right) & l_{2}^{2}h_{2}+\left(l_{1}l_{2}h_{2}\right)cos\left(\theta_{2}\left(t\right)\right)\\ l_{2}^{2}h_{2}+\left(l_{1}l_{2}h_{2}\right)cos\left(\theta_{2}\left(t\right)\right) & l_{2}^{2}h_{2} \end{array}\right],\\ C\left(\theta \left(t\right),\dot{\theta }\left(t\right)\right)\dot{\theta }\left(t\right) & =\left[\begin{array}{c} -\left(h_{2}l_{1}l_{2}\right)sin\left(\theta_{2}\left(t\right)\right)\left\{{\dot{\theta }}_{2}^{2}\left(t\right)+2{\dot{\theta }}_{1}\left(t\right){\dot{\theta }}_{2}\left(t\right)\right\}\\ \left(h_{2}l_{1}l_{2}\right)sin\left(\theta_{2}\left(t\right)\right){\dot{\theta }}_{2}^{2}\left(t\right) \end{array}\right],\\ G(\theta (t)) & =\left[\begin{array}{c} h_{1}l_{1}gcos\left(\theta_{1}\left(t\right)\right)+h_{2}g(l_{1}cos\left(\theta_{1}\left(t\right)\right)+l_{2}cos\left(\theta_{1}\left(t\right)+\theta_{2}\left(t\right)\right)\\ h_{2}l_{2}gcos\left(\theta_{1}\left(t\right)+\theta_{2}\left(t\right)\right) \end{array}\right],\\ F(\dot{\theta }\left(t\right)) & =\left[\begin{array}{c} f_{c1}sgn\left({\dot{\theta }}_{1}\left(t\right)+f_{v1}{\dot{\theta }}_{1}\left(t\right)\right)\\ f_{c2}sgn\left({\dot{\theta }}_{2}\left(t\right)+f_{v2}{\dot{\theta }}_{2}\left(t\right)\right) \end{array}\right], \end{array}$$

where $\theta \left(t\right)={\left[\begin{array}{cc} \theta_{1}\left(t\right) & \theta_{2}\left(t\right) \end{array}\right]}^{T}$, and $\theta_{i}\left(t\right)$ represents the joint position of *i*-th joint. The simulation model and parameters are adopted from [8] for consistency and comparability. In the system matrices (24), scalars are defined as $h_{1}=9$ [kg], $h_{2}=6$ [kg], $l_{1}=0.4$ [m], $l_{2}=0.2$ [m], and g=9.81 [m/s^2^]. The friction coefficients are $f_{v1}=10$ [N· m · s], $f_{c1}=10$ [N· m · s], $f_{v2}=10$ [N· m · s], and $f_{c2}=10$ [N· m · s]. The parameters used in the control torque are configured as L=0.001 [s], $\ell_{1}=30$, $\ell_{2}=5$, $\bar{H}=diag\left\{0.08,0.04\right\}$, and α=0.3. The unpredictable disturbance is described by $\tau_{d}\left(t\right)=10cos\left(2\pi t\right)$. The desired position is given by $\theta_{d}\left(t\right)={\left[\begin{array}{cc} 4sin(3t) & 3sin(2t) \end{array}\right]}^{T}$. We set the adjustable parameters of the RBFNN as $M_{1}=10\cdot J_{1,3}$, $M_{2}=8\cdot J_{1,3}$, $M_{3}=6\cdot J_{1,3}$, $M_{4}=4\cdot J_{1,3}$, $M_{5}=2\cdot J_{1,3}$, $M_{6}=0\cdot J_{1,3}$, $M_{7}=-M_{5}$, $M_{8}=-M_{4}$, $M_{9}=-M_{3}$, $M_{10}=-M_{2}$, $M_{11}=-M_{1}$, η=5, $\zeta_{1}=\zeta_{2}=1$, $\gamma_{1}=0.03$, $\gamma_{2}=0.04$, b=0.3. The parameters of the quasi-convex function, ρ and λ, are set to 10 and 0.0213, respectively. For effective comparisons of control performance, this paper contains the results of the ASMC (12) using the convex function-based gain in [13], the proposed ASMC (13), and its specific case when $\Xi \left(t\right)=\alpha I_{2}$. The parameters of the convex function-based gain, $\rho_{2}$ and $\lambda_{2}$, are set to 5 and 0.05, respectively.

### 4.2. Simulation Results

**Figure 3:**
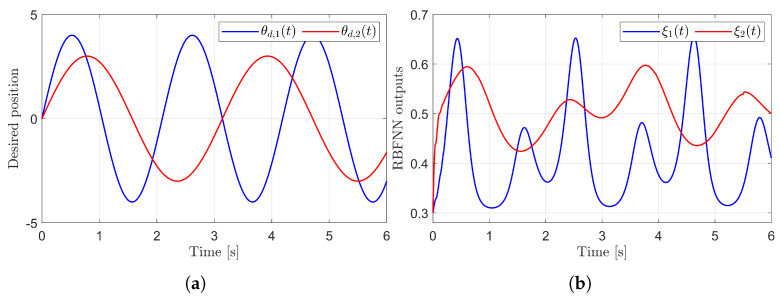
Desired position trajectories and associated RBFNN outputs. (**a**) Desired positions. (**b**) RBFNN outputs.

**Figure 4:**
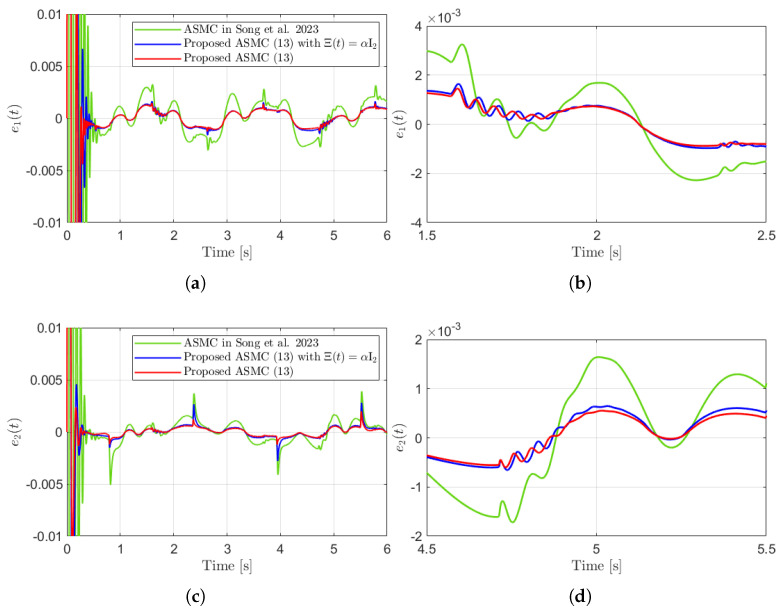
Tracking errors and their enlarged views in the simulation compared with [13] (Song et al., 2023). (**a**) $e_{1}\left(t\right)$. (**b**) Enlarged view of $e_{1}\left(t\right)$. (**c**) $e_{2}\left(t\right)$. (**d**) Enlarged view of $e_{2}\left(t\right)$.

**Figure 5:**
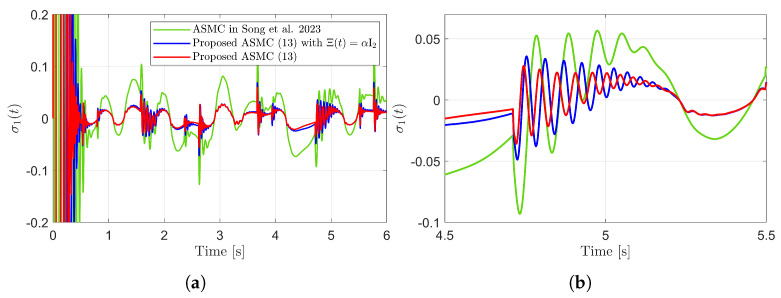

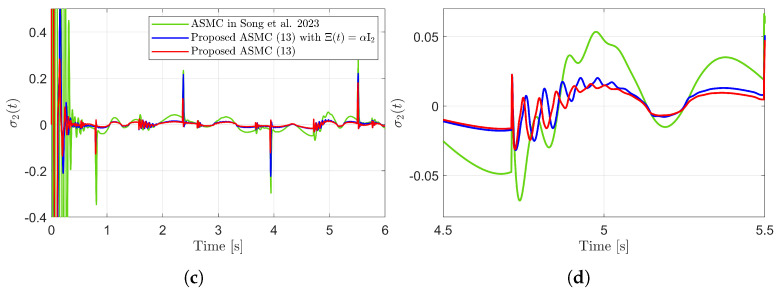
Sliding variables and their enlarged views in the simulation compared with [13] (Song et al., 2023). (**a**) $\sigma_{1}\left(t\right)$. (**b**) Enlarged view of $\sigma_{1}\left(t\right)$. (**c**) $\sigma_{2}\left(t\right)$. (**d**) Enlarged view of $\sigma_{2}\left(t\right)$.

**Figure 6:**
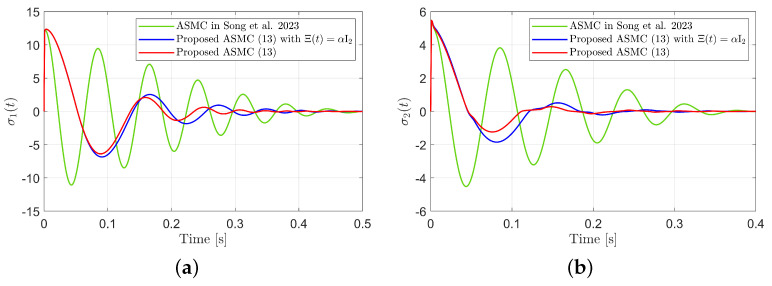
Sliding variables during reaching phase in the simulation compared with [13] (Song et al., 2023). (**a**) $\sigma_{2}\left(t\right)$. (**b**) $\sigma_{2}\left(t\right)$.

**Figure 7:**
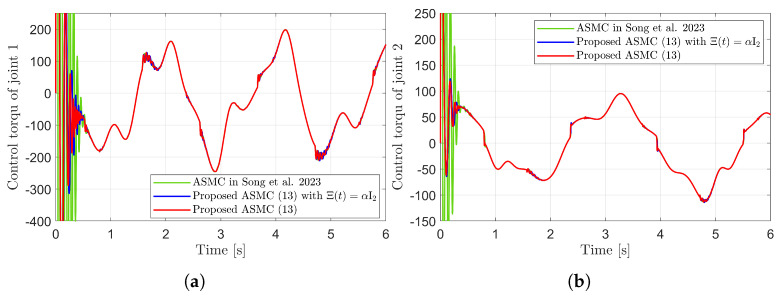
Control torques in the simulation compared with [13] (Song et al., 2023). (**a**) Control torque of joint 1. (**b**) Control torque of joint 2.

**Figure 8:**
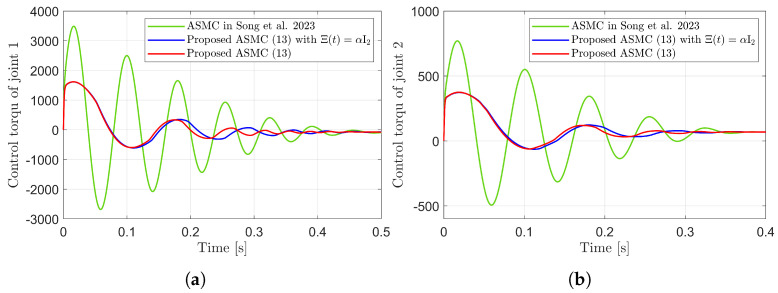
Control torques during reaching phase in the simulation compared with [13] (Song et al., 2023). (**a**) Control torque of joint 1. (**b**) Control torque of joint 2.

Figure 3 presents the desired position trajectories and associated RBFNN outputs which are elements of the estimated matrix Ξ(t). From Figure 3, it can be shown that the weight update law in (17) effectively prevents the RBFNN output from diverging. Figure 4 and Figure 5 illustrate that employing the estimated matrix Ξ(t) significantly reduces tracking errors compared to the case using a constant parameter $\alpha I_{2}$ in the control input (13). In addition, Figure 4 and Figure 5 compare tracking performance of the proposed ASMC (13) when $\Xi \left(t\right)=\alpha I_{2}$ with the ASMC (12) using the convex function-based gain in [13]. Notably, Figure 6 reveals that the convex function-based ASMC induces more frequent oscillations during the reaching phase. Additionally, the rapid convergence of the sliding variable is not only essential for improving tracking accuracy and disturbance rejection, but also plays a critical role in ensuring reliable and real-time control in practical systems, as emphasized in [23,24]. The reduced chattering phenomenon in the proposed control torque is clearly observed in Figure 7 and Figure 8.

## 5. Experiment

### 5.1. Experiment Setup

**Figure 9:**
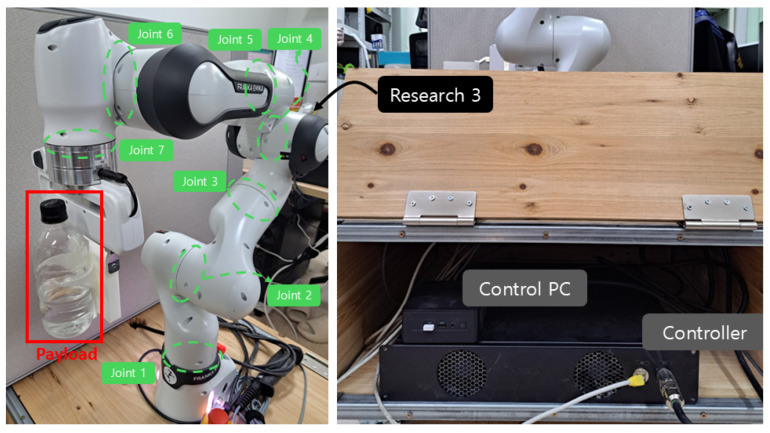
Franka Research 3 and control environments.

Figure 9 illustrates the 7-joint robotic manipulator, Franka Research 3, used in the experiment. The proposed controller was implemented using MATLAB/Simulink R2023b and executed on an Ubuntu 20.04 system. Real-time communication was established between MATLAB and the Franka Research 3 using the Franka Control Interface, enabling direct torque commands to be sent at each control cycle with a sampling rate of 1 kHz. Joint positions and velocities were measured using the internal encoders, while joint torques were obtained from the built-in torque sensors. The attached payload is 0.5 [kg], including the 0.338 [kg] weight of the water bottle. The attached payload generates highly irregular disturbances due to the movement of the water. Three control methods, referred as ASMC (12) using the convex function-based gain in [13], the proposed ASMC (13), and its specific case when $\Xi \left(t\right)=\alpha I_{7}$, are compared in the experiment. The initial pose of Franka Research 3 is $\theta_{0}={\left[\begin{array}{ccccccc} 0 & -0.7854 & 0 & -2.3562 & 0 & 1.5708 & 0.7854 \end{array}\right]}^{T}$ and the desired position is set as follows.

(25) $$\begin{array}{c} \theta_{d}\left(t\right)={\left[\begin{array}{ccccccc} \frac{0.7}{\pi }cos\left(\pi t\right) & -\frac{1.5}{\pi }cos\left(\frac{2}{3}\pi t\right) & \frac{1.5}{\pi }cos\left(\frac{2}{3}\pi t\right) & -\frac{0.7}{\pi }cos\left(\pi t\right) & 0 & 0 & 0 \end{array}\right]}^{T}+\theta_{0}. \end{array}$$

The center $M_{j}$ and the width η of the RBFNN are chosen to be the same as those used in the simulation. The control parameters are set as follows. $\bar{H}=0.01I_{7}$, $\ell_{1}=\ell_{2}=5I_{7}$, L=0.001 [s], $\zeta_{1}=\zeta_{2}=\zeta_{3}=\zeta_{4}=\zeta_{5}=\zeta_{6}=\zeta_{7}=1$, $\gamma_{1}=\gamma_{2}=\gamma_{3}=\gamma_{4}=0.4$, $\gamma_{5}=\gamma_{6}=\gamma_{7}=0.2$, α=b=0.4. The parameters of the quasi-convex function-based gain (18), ρ and λ, are set to 10 and 0.255, respectively. The parameters of the convex function-based gain in [13], $\rho_{2}$ and $\lambda_{2}$, are set to 3 and 0.5, respectively.

### 5.2. Experiment Results

**Figure 10:**
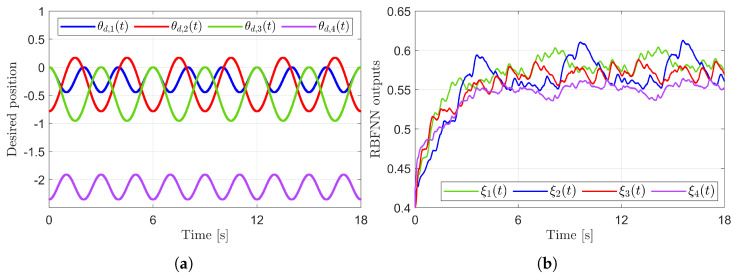
Desired position trajectories and associated RBFNN outputs. (**a**) Desired position trajectories. (**b**) RBFNN outputs.

**Figure 11:**
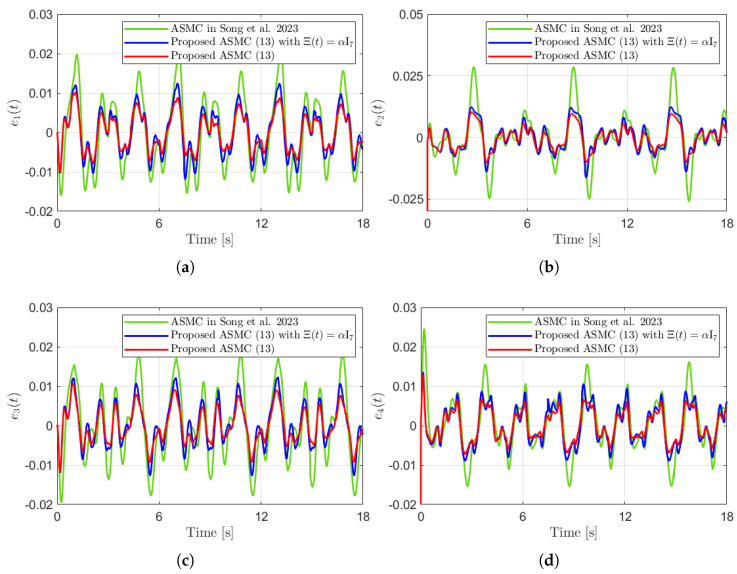
Tracking errors in the experiment compared with [13] (Song et al., 2023). (**a**) $e_{1}\left(t\right)$. (**b**) $e_{2}\left(t\right)$. (**c**) $e_{3}\left(t\right)$. (**d**) $e_{4}\left(t\right)$.

**Figure 12:**
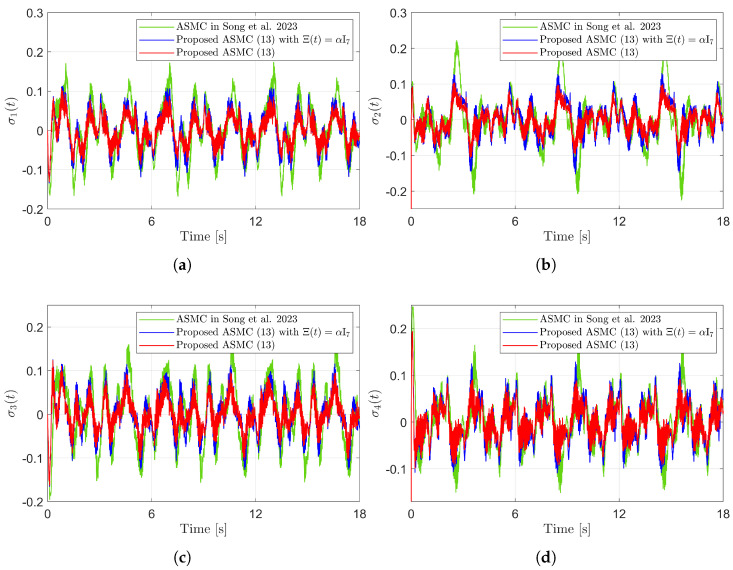
Sliding variables in the experiment compared with [13] (Song et al., 2023). (**a**) $\sigma_{1}\left(t\right)$. (**b**) $\sigma_{2}\left(t\right)$. (**c**) $\sigma_{3}\left(t\right)$. (**d**) $\sigma_{4}\left(t\right)$.

**Figure 13:**
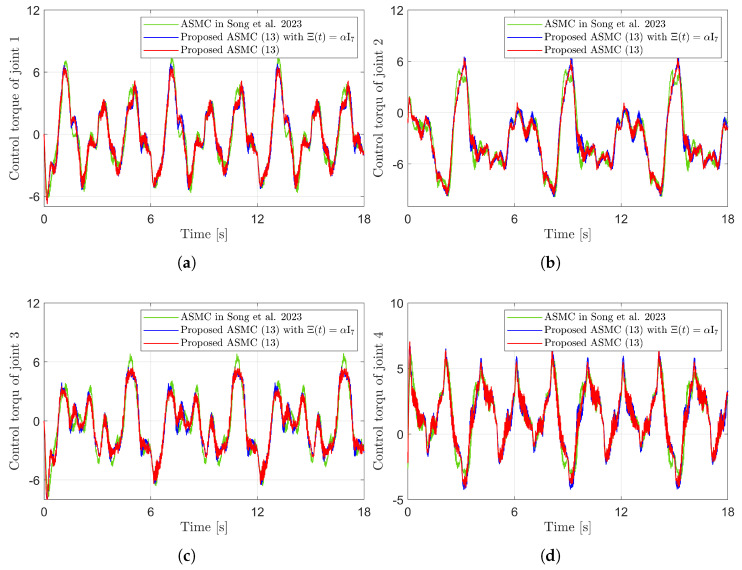
Comparison of control torques in the experiment compared with [13] (Song et al., 2023). (**a**) Control torque of joint 1. (**b**) Control torque of joint 2. (**c**) Control torque of joint 3. (**d**) Control torque of joint 4.

Figure 10 presents the desired position trajectories along with the corresponding RBFNN outputs, which constitute the estimated matrix Ξ(t). It is observed that the weight update law in (17) effectively prevents the RBFNN output from diverging, even under real environments. Figure 11 and Figure 12 demonstrate that using the estimated Ξ(t) leads to reduced tracking errors compared to the case using a fixed parameter $\alpha I_{7}$ in the control input (13). Furthermore, Figure 11 and Figure 12 compare the tracking performance of the proposed ASMC when $\Xi \left(t\right)=\alpha I_{7}$ with the ASMC (12) using the convex function-based gain in [13], showing that the proposed function contributes improved tracking performance. Notably, Figure 12 and Figure 13 reveal improved tracking performance with reduced chattering phenomenon of the proposed control torque (13).

**Remark** **4.** *In addition to the simulation, the effectiveness of the proposed control method is verified through an experiment using a real 7-joint robotic manipulator, Franka Research 3. The proposed control method is tested with highly irregular disturbances caused by a water-filled payload during fast motion. The results demonstrate that the proposed control method is not only theoretically sound but also practical and robust for robotic manipulator applications in real environments.*

## 6. Conclusions

This paper proposed the ASMC strategy tailored for robotic manipulators, featuring a quasi-convex function-based control gain and the TDE enhanced by NNs. To compensate for TDE errors, the proposed method utilized both the previous TDE error and the RBFNN with a weight update law that includes a damping term to prevent divergence. Additionally, a continuous gain that is quasi-convex function is proposed to replace the traditional switching control gain. This function preserved the continuous gain and smooth transitions between convex and concave characteristics depending on the magnitude of the sliding variable. As a result, the proposed gain effectively suppressed the chattering phenomenon caused by abrupt changes in the SMC gain and mitigated the overestimation problem associated with the convex function. The stability of the proposed control method is guaranteed in the sense of uniform ultimate boundedness, and its effectiveness was validated through both simulation and experiment results. In future work, the proposed ASMC method will be extended to complex robot systems.

## Data Availability

Data are contained within the article.
